# T Helper 17 Promotes Induction of Antigen-Specific Gut-Mucosal Cytotoxic T Lymphocytes following Adenovirus Vector Vaccination

**DOI:** 10.3389/fimmu.2017.01456

**Published:** 2017-11-06

**Authors:** Masahisa Hemmi, Masashi Tachibana, Natsuki Fujimoto, Masaki Shoji, Fuminori Sakurai, Kouji Kobiyama, Ken J. Ishii, Shizuo Akira, Hiroyuki Mizuguchi

**Affiliations:** ^1^Laboratory of Biochemistry and Molecular Biology, Graduate School of Pharmaceutical Sciences, Osaka University, Osaka, Japan; ^2^Laboratory of Biotechnology and Therapeutics, Graduate School of Pharmaceutical Sciences, Osaka University, Osaka, Japan; ^3^Global Center for Medical Engineering and Informatics, Osaka University, Osaka, Japan; ^4^Laboratory of Regulatory Sciences for Oligonucleotide Therapeutics, Clinical Drug Development Unit, Graduate School of Pharmaceutical Sciences, Osaka University, Osaka, Japan; ^5^Center for Vaccine and Adjuvant Research, National Institutes of Biomedical Innovation, Health and Nutrition, Osaka, Japan; ^6^Laboratory of Vaccine Science, World Premier International Research Center Immunology Frontier Research Center, Osaka University, Osaka, Japan; ^7^Laboratory of Host Defense, World Premier International Research Center Immunology Frontier Research Center, Osaka University, Osaka, Japan; ^8^Department of Host Defense, The Research Institute for Microbial Diseases, Osaka University, Osaka, Japan; ^9^iPS Cell-Based Research Project on Hepatic Toxicity and Metabolism, Graduate School of Pharmaceutical Sciences, Osaka University, Osaka, Japan; ^10^Laboratory of Hepatocyte Regulation, National Institutes of Biomedical Innovation, Health and Nutrition, Osaka, Japan

**Keywords:** T helper 17 cells, cytotoxic T lymphocyte, vaccines, DNA vaccines, mucosal immunity, gut mucosa, type I interferon, innate immunity

## Abstract

Few current vaccines can establish antigen (Ag)-specific immune responses in both mucosal and systemic compartments. Therefore, development of vaccines providing defense against diverse infectious agents in both compartments is of high priority in global health. Intramuscular vaccination of an adenovirus vector (Adv) has been shown to induce Ag-specific cytotoxic T lymphocytes (CTLs) in both systemic and gut-mucosal compartments. We previously found that type I interferon (IFN) signaling is required for induction of gut-mucosal, but not systemic, CTLs following vaccination; however, the molecular mechanism involving type I IFN signaling remains unknown. Here, we found that T helper 17 (Th17)-polarizing cytokine expression was down-regulated in the inguinal lymph nodes (iLNs) of *Ifnar2^−/−^* mice, resulting in the reduction of Ag-specific Th17 cells in the iLNs and gut mucosa of the mice. We also found that prior transfer of Th17 cells reversed the decrease in the number of Ag-specific gut-mucosal CTLs in *Ifnar2^−/−^* mice following Adv vaccination. Additionally, prior transfer of Th17 cells into wild-type mice enhanced the induction of Ag-specific CTLs in the gut mucosa, but not in systemic compartments, suggesting a gut mucosa-specific mechanism where Th17 cells regulate the magnitude of vaccine-elicited Ag-specific CTL responses. These data suggest that Th17 cells translate systemic type I IFN signaling into a gut-mucosal CTL response following vaccination, which could promote the development of promising Adv vaccines capable of establishing both systemic and gut-mucosal protective immunity.

## Introduction

Most infectious agents enter the body *via* the extensive surface areas of mucosal membranes; therefore, development of vaccines capable of establishing protective immunity in both mucosal and systemic compartments is a high-priority global health issue (1, 2). However, few vaccines in current use can establish antigen (Ag)-specific immune responses at both sites (3), and induction of mucosal immunity by systemic vaccination is challenging because of the distinct immunological characteristics of the mucosal immune system (3).

Replication-incompetent recombinant adenovirus vectors (Advs) have advantages as gene therapy vectors. They provide the highest gene transduction efficiency among currently available vectors, exhibit low levels of genotoxicity because they are not integrated into chromosomal DNA, and can be easily prepared at high titers. Therefore, Adv is among the most promising vectors for gene therapy. Moreover, Adv can be used as a gene therapy-based vaccine and has been utilized in preclinical and clinical vaccine research (4–7). Previous reports showed that intramuscular (*i.m*.) immunization with Adv vaccines expressing simian immunodeficiency virus (SIV) proteins induced protective and durable SIV Ag-specific cytotoxic T lymphocytes (CTLs) in both gut-mucosal and systemic sites, in mice and rhesus macaques (8–10). Since Adv vaccination achieves high-viral clearance, mucosal CTL induction is considered important for effective vaccines (10). Therefore, Adv vaccines are expected to serve as next-generation mucosal vaccines capable of combating severe intracellular pathogens; however, the development of efficacious Adv vaccines has yet to be achieved. To generate more protective Adv vaccines, it is necessary to identify the mechanisms involved in Adv-vaccine establishment of both systemic and gut-mucosal immunity.

Innate immune responses are indispensable for the optimum induction of adaptive immune responses (11–13). Moreover, accumulating evidence shows that adjuvants that activate the innate immune response are important for inducing the effects of vaccines (14, 15). Several reports show that Adv-derived nucleic acids, such as adenoviral genomic DNA and non-coding RNA, activate the innate immune response, and induce the production of innate immune cytokines (16–20), indicating that the adenoviral components of Adv vaccines serve as built-in adjuvants. These Adv-derived nucleic acids trigger innate immune responses through several pathways, resulting in robust production of type I interferons (IFNs) and pro-inflammatory cytokines. Type I IFNs induced by Adv immunization play an important role in subsequent systemic adaptive immunity. Both dendritic cells (DCs) and other cell types, such as stromal cells, produce type I IFNs *in vivo* and are involved in the induction of adaptive immune response (20–22). Moreover, type I IFN signaling is required for efficient humoral immunity after intravenous Adv immunization (23), suggesting that type I IFN signaling controls the efficacy of Adv vaccines. Therefore, determining the role of type I IFN signaling *in vivo* is important for the development of efficacious Adv vaccines. We previously reported that type I IFN signaling following *i.m*. Adv vaccination is required for induction of Ag-specific CTLs in the gut-mucosal site, but not in the systemic site (24). This finding indicates that type I IFN signaling plays important roles in positive regulation of Ag-specific gut-mucosal cellular immunity; however, it remains unclear how Adv-induced type I IFN signaling translates systemic innate immunity into gut-mucosal adaptive immunity.

In the present study, we used type I IFN-receptor-deficient (*Ifnar2^−/−^*) mice to investigate the physiological role of type I IFN signaling. Our results showed that type I IFN signaling was indispensable for induction of Ag-specific T helper 17 (Th17) cells in the gut mucosa following *i.m*. Adv vaccination, and that Th17 cells promoted the induction of Ag-specific CTLs in the gut mucosa. These data suggest that Th17 cells translate systemic type I IFN signaling into a gut-mucosal CTL response following Adv vaccination. Our findings promote the development of safer and more efficient Adv vaccines capable of establishing protective and durable immunity in both systemic and gut-mucosal compartments.

## Materials and Methods

### Ethics Statement

All animal-based experimental procedures used in this study were performed in accordance with the institutional guidelines for animal experiments at Osaka University (Douyaku 28-3-1) and the National Institutes of Biomedical Innovation, Health and Nutrition (DS19-106).

### Mice

C57BL/6J wild-type (WT) (Japan SLC, Hamamatsu, Japan) and *Ifnar2^−/−^* mice (C57BL/6J background) were prepared as described previously (24, 25). All mice were bred in an animal facility under specific pathogen-free conditions, and female mice were used for experiments between 6 and 8 weeks of age.

### Adv Production and Immunization

β-galactosidase, encoded by *LacZ*, was used as a model Ag. The adenovirus type 5 vector expressing LacZ (Ad-LacZ) was constructed as described previously (26, 27). Briefly, the expression cassette containing the chicken β-actin (*Actb*) promoter with the cytomegalovirus enhancer-driven (28) *LacZ* gene was inserted into the E1-deletion region of the E1/E3-deleted adenovirus type 5 genome. This virus was propagated in HEK293 cells and then purified using standard techniques. Determination of the virus particle (vp) titers was accomplished spectrophotometrically according to the methods of Maizel et al. (29). All mice were injected under anesthesia in the both quadriceps muscles with Ad-LacZ at 10^10^ vp/mouse (5 × 10^9^ vp/50 μL PBS/muscle).

### Isolation of Mononuclear Cells

The spleen and lymph nodes were dissected and pressed through a 70-µm cell strainer (Corning, Corning, NY, USA), and cells were washed with 2% FCS/PBS. Splenocyte isolation was followed by the lysis of red blood cells. Small intestinal lamina propria (LP) cells were isolated using a standard enzymatic dissociation procedure as described previously (30). Briefly, small intestines were removed from Peyer’s patches and cut open longitudinally. After washing with PBS, the tissues were cut into small pieces and stirred in RPMI 1640 supplemented with 2% FCS and 0.5 mM EDTA at 37°C for 20 min. The specimens were washed again with RPMI 1640 supplemented 2% FCS and then minced and digested twice in RPMI 1640 supplemented with 10% FCS and 0.5 mg/mL collagenase (Wako Pure Chemical Industries, Osaka, Japan) at 37°C for 30 min with stirring. Mononuclear cells were then isolated by a discontinuous density gradient procedure (40 and 75%) with Percoll PLUS (GE Healthcare, Little Chalfont, UK). The cells that were layered between the 40 and 75% interfaces were collected as small intestinal LP lymphocytes. Muscle cells were minced and then digested in RPMI 1640 supplemented with 10% FCS and 1.0 mg/mL collagenase D (Roche, Basel, Switzerland) at 37°C for 30 min with stirring. The supernatant was used as the source of mononuclear cells.

### RNA Isolation and Quantitative Reverse Transcription-Polymerase Chain Reaction

Total RNA isolation and cDNA synthesis from mononuclear cells or whole muscles were performed as described previously (27). The mRNA level of each gene was normalized against that of *Actb* or glyceraldehyde 3-phosphate dehydrogenase *(Gapdh)*. The primer sequences used in this study were: *Actb* forward, 5′-CCTATGTGTCATTTGGGTGGATG-3′; *Actb* reverse, 5′-GGTTGTCAGGGGAGTGTTGAT-3′; *Gapdh* forward, 5′-CCAGGTTGTCTCCTGCGACTT-3′; *Gapdh* reverse, 5′-CCTGTTGCTGTAGCCGTATTCA-3′; *Il12b* forward, 5′-CCGCAACAACGCCATCTATG-3′; *Il12b* reverse, 5′-CCCGAATGTCTGACGTATTGAAG-3′; *Il2* forward, 5′-TGAGCAGGATGGAGAATTACAGG-3′; *Il2* reverse, 5′-GTCCAAGTTCATCTTCTAGGCAC-3′; *Tgfb* forward, 5′-TCGTTTGACCACAGTCCCTAA-3′; *Tgfb* reverse, 5′-GAAGTCGAAAGTACAGGCTGTTT-3′; *Il1b* forward, 5′-CACACTGCTGGTCATCAAGAT-3′; *Il1b* reverse, 5′-TCACTCCTGTAATACTGGAGGC-3′; *Il6* forward, 5′-CCTCTACCAAAACCATTCGGAG-3′; *Il6* reverse, 5′-CTGTCCACGTACAATTCGTTCA-3′; *Il23a* forward, 5′-AGTTGTGCTGAGCTGTATGGA-3′; *Il23a* reverse, 5′-CGGCTGCTTGAAGTAAAACAGG-3′; *Il17a* forward, 5′-CTCCAGAAGGCCCTCAGACTAC-3′; *Il17a* reverse, 5′-GGGTCTTCATTGCGGTGG-3′; *Il17f* forward, 5′-CCCATGGGATTACAACATCACTC-3′; *Il17f* reverse, 5′-CACTGGGCCTCAGCGATC-3′; *Il22* forward, 5′-ATGAGTTTTTCCCTTATGGGGAC-3′; *Il22* reverse, 5′-GCTGGAAGTTGGACACCTCAA-3′; *Ccl2* forward, 5′-GTTGGCTCAGCCAGATGCA-3′; *Ccl2* reverse, 5′-AGCCTACTCATTGGGATCATCTTG-3′; *Ccl7* forward, 5′-AGCTACAGAAGGATCACCAG-3′; *Ccl7* reverse, 5′-CACATTCCTACAGACAGCTC-3′.

### Flow Cytometry

The anti-mouse antibodies (Abs) used in this study were purchased from eBioscience [APC-RORγt (B2D) and eFluor 450-Foxp3 (FJK-16s); Thermo Fisher Scientific, Waltham, MA, USA], BioLegend [Purified-CD16/32 (93), Brilliant Violet 421-Streptavidin, APC-Cy7- and PE-Cy7-CD3ε (145-2C11), FITC-, PE-, and PerCP-Cy5.5-CD4 (RM4-5), APC-Cy7-CD8α (53-6.7), Biotin- and PE-Cy7-CD11b (M1/70), APC- and PE-CD11c (N418), APC-CD25 (PC61), FITC-CD45 (30F-11), Pacific Blue-CD62L (MEL-14), PerCP-Cy5.5-Ly-6C (HK1.4), APC-Ly-6G (2A8), FITC-I-Ab (AF6-120.1), Pacific Blue-CD80 (16-10A1), APC-CD86 (GL-1), Alexa Fluor 647-CCR9 (CW-1.2), Biotin-α_4_β_7_ integrin (DATK32), FITC-T-bet (4B10), Alexa Fluor 488-Foxp3 (FJK-16s), PE-Cy7-IFN-γ (XMG1.2), PE-IL-17A (TC11-18H10.1); San Diego, CA, USA], Bio-Rad [FITC- and Pacific Blue-CD8α (KT15); Hercules, CA, USA], R&D Systems [PE-CCR2 (475301); Minneapolis, MN, USA], and BD Biosciences [Brilliant Violet 421-RORγt (Q31-378); San Jose, CA, USA]. β-gal-specific CTLs were stained with PE-H-2K^b^/β-gal_96-103_ (DAPIYTNV) tetramer reagent (MBL, Nagoya, Japan) according to the manufacturer’s protocols. Cells were incubated with anti-CD16/32 Ab, followed by staining with fluorescence-conjugated Abs. Dead cells were excluded by staining with 7-amino-actinomycin D (eBioscience), SYTOX-Blue (Invitrogen), or LIVE/DEAD Fixable Blue Dead Cell Stain kit (Invitrogen). Samples were acquired using a BD LSRFortessa flow cytometer (BD Biosciences) or a MACSQuant flow cytometer (Miltenyi Biotec, Bergisch Gladbach, Germany), and samples were analyzed with BD FACSDiva software (BD Biosciences) or FlowJo software (TreeStar Inc., Ashland, OR, USA).

### Detection of β-Gal-Specific Th17 Cells

After isolation of mononuclear cells, CD4^+^ T cells were purified using PE anti-CD4, followed by anti-PE ultrapure microbeads (Miltenyi Biotec), and sorted using the AutoMACS Pro sorter (Miltenyi Biotec). Splenic DCs were purified by using PE anti-CD11c, followed by anti-PE ultrapure microbeads, and sorted as described above. CD4^+^ T cells were co-cultured with splenic DCs (CD4^+^ T cells:splenic DCs = 4:1) in the presence of 100 µg/mL β-galactosidase (Sigma-Aldrich, St. Louis, MO, USA) for 4 days. IL-17A and IL-22 in the supernatant were detected by enzyme-linked immunosorbent assay kits (R&D systems) according to the manufacturer’s instructions.

### *In Vitro* Adv Infection of Bone-Marrow-Derived DCs (BMDCs)

Murine BMDCs were prepared as described previously (31) and infected with 10^4^ vp/cell Ad-LacZ for 24 h.

### Co-culture of CD4^+^ T Cells with Adv-Infected BMDCs

Adenovirus vector-infected BMDCs were prepared as described, and CD4^+^ T cells enriched by negative selection from spleens and lymph nodes were co-cultured with Adv-infected BMDCs (CD4^+^ T cells:BMDCs = 4:1) for 8 days. After co-culture, cells were stained to detect intracellular cytokines or transcription factors.

### Intracellular Staining

For intracellular cytokine staining, a BD Cytofix/Cytoperm kit (BD Biosciences) was used according to the manufacturer’s instructions. Briefly, cells were stimulated with 20 ng/mL PMA and 500 ng/mL ionomycin in the presence of BD GolgiStop (BD Biosciences) for 6 h. After stimulation, the cells were collected and subjected to dead cell and surface staining, fixed, and permeabilized with BD Cytofix/Cytoperm solution, and stained for intracellular IL-17A and IFN-γ. For intracellular transcription factor staining, the Foxp3/transcription factor staining buffer set (eBioscience) was used according to the manufacturer’s instructions. Briefly, cells were stained for dead cells and surface molecules, fixed, and permeabilized with Foxp3 fixation/permeabilization solution, and stained for intracellular T-bet, Foxp3, and RORγt.

### *In Vitro* Th17 Differentiation and Cell Transfer

Naïve CD4^+^CD25^-^CD62L^+^ cells were sorted from spleens and lymph nodes using an SH800 cell sorter (Sony Biotechnology, Tokyo, Japan). Naïve CD4^+^ T cells were cultured for 4 days with Iscove’s modified Dulbecco’s medium (Sigma-Aldrich) supplemented with 10% FCS, 2 mM GlutaMAX (Gibco; Thermo Fisher Scientific), 100 U/mL penicillin, 100 µg/mL streptomycin, and 50 µM 2-mercaptoethanol in the presence of 2 µg/mL plate-bound anti-CD3ε, 2 µg/mL soluble anti-CD28, 10 µg/mL anti-IFN-γ, anti-IL-4, 20 ng/mL IL-1β (Peprotech), IL-6, and IL-23 (R&D systems). Differentiated cells were stained using the intracellular staining protocol, and the frequency of Th17 cells was determined according to the expression of IL-17A and RORγt as determined by flow cytometry. Differentiated cells (2 × 10^6^ cells/mouse) were transferred into mice through the tail vein.

### Statistical Analysis

All results are shown as the mean ± SEM (biological replicates) or SD (technical replicates). Statistical significance was analyzed using GraphPad Prism (GraphPad Software, San Diego, CA, USA) and a two-tailed Student’s *t*-test between two groups, one-way analysis of variance, or a Kruskal–Wallis test for more than three groups. *p* < 0.05 indicated a significant difference.

## Results

### Type I IFN Signaling Is Dispensable for the Induction of Gut-Homing CTLs in the Inguinal Lymph Nodes (iLNs)

**Figure 1:**
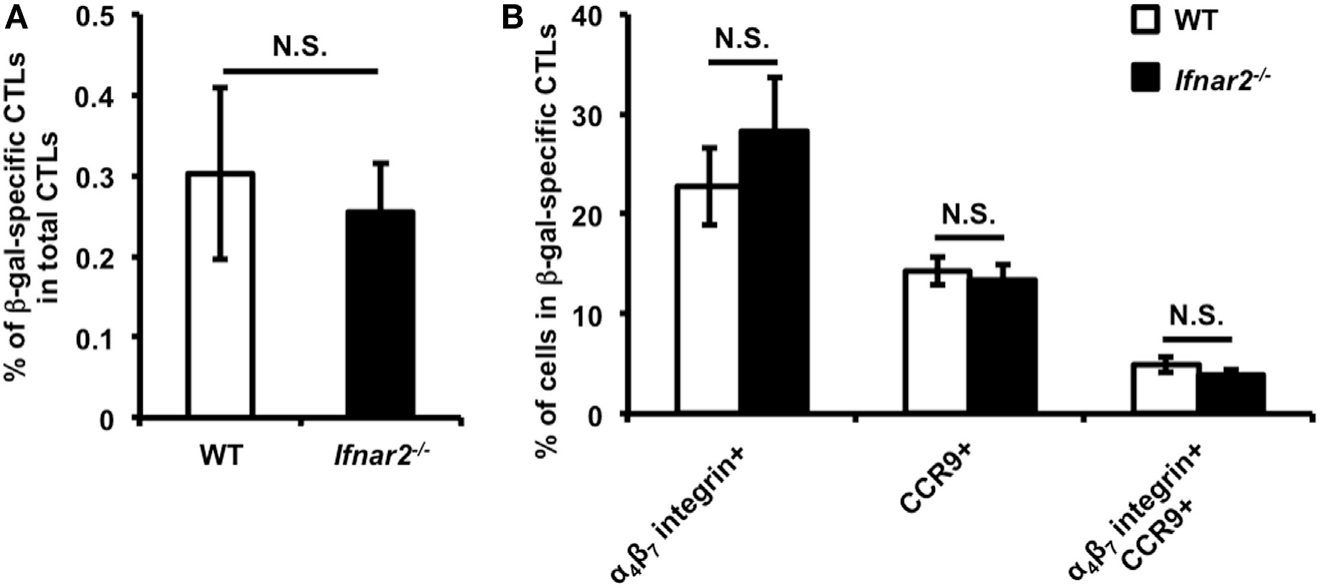
Priming of CD8^+^ T cells and imprinting of gut-homing capacity on these cells is equally induced in the inguinal lymph nodes (iLNs) of wild-type and *Ifnar2^−/−^* mice following intramuscular (*i.m*.) adenovirus vector vaccination. At 7 days after *i.m*. Ad-LacZ vaccination, the frequencies of **(A)** β-gal-specific CD8^+^ T cells in the CD8^+^ T cell population and **(B)** gut-homing-molecule-expressing cells in β-gal-specific CD8^+^ T cells in iLNs were measured by flow cytometry. Data are the pools of two independent experiments and are shown as the mean ± SEM (*n* = 6). N.S., not significant (according to Student’s *t*-test).

After *i.m*. Adv vaccination, T cell priming occurred in iLNs acting as draining lymph nodes for the vaccination sites, resulting in the induction of Ag-specific CTLs. First, we determined whether type I IFN signaling had a direct effect on induction of Ag-specific CTLs in iLNs. We confirmed that the frequencies of Ag-specific CTLs among total CTLs in iLNs were similar in WT and *Ifnar2^−/−^* mice (Figure 1A), suggesting that T cell priming was equally induced in these mice. Previous reports showed that some Ag-specific CTLs in iLNs migrated into the gut-mucosal compartment by expressing the gut-homing molecules C–C chemokine receptor (CCR)9 and α_4_β_7_ integrin (8, 32). To determine whether type I IFN signaling is involved in the induction of gut-homing Ag-specific CTLs in iLNs, we examined the frequencies of gut-homing CTLs in Ag-specific CTL populations. The frequencies of CCR9^+^α_4_β_7_ integrin^−^, CCR9^−^α_4_β_7_ integrin^+^, and CCR9^+^α_4_β_7_ integrin^+^ CTLs were similar in WT and *Ifnar2^−/−^* mice (Figure 1B). These results suggest that type I IFN signaling was dispensable for the induction of Ag-specific CTLs and the imprinting of gut-homing capabilities on those CTLs in iLNs, and also indicated that type I IFN signaling regulated the induction of gut-mucosal CTLs through other mechanisms.

### Type I IFN Signaling Is Required for the Expression of Th17-Polarizing Cytokines

**Figure 2:**
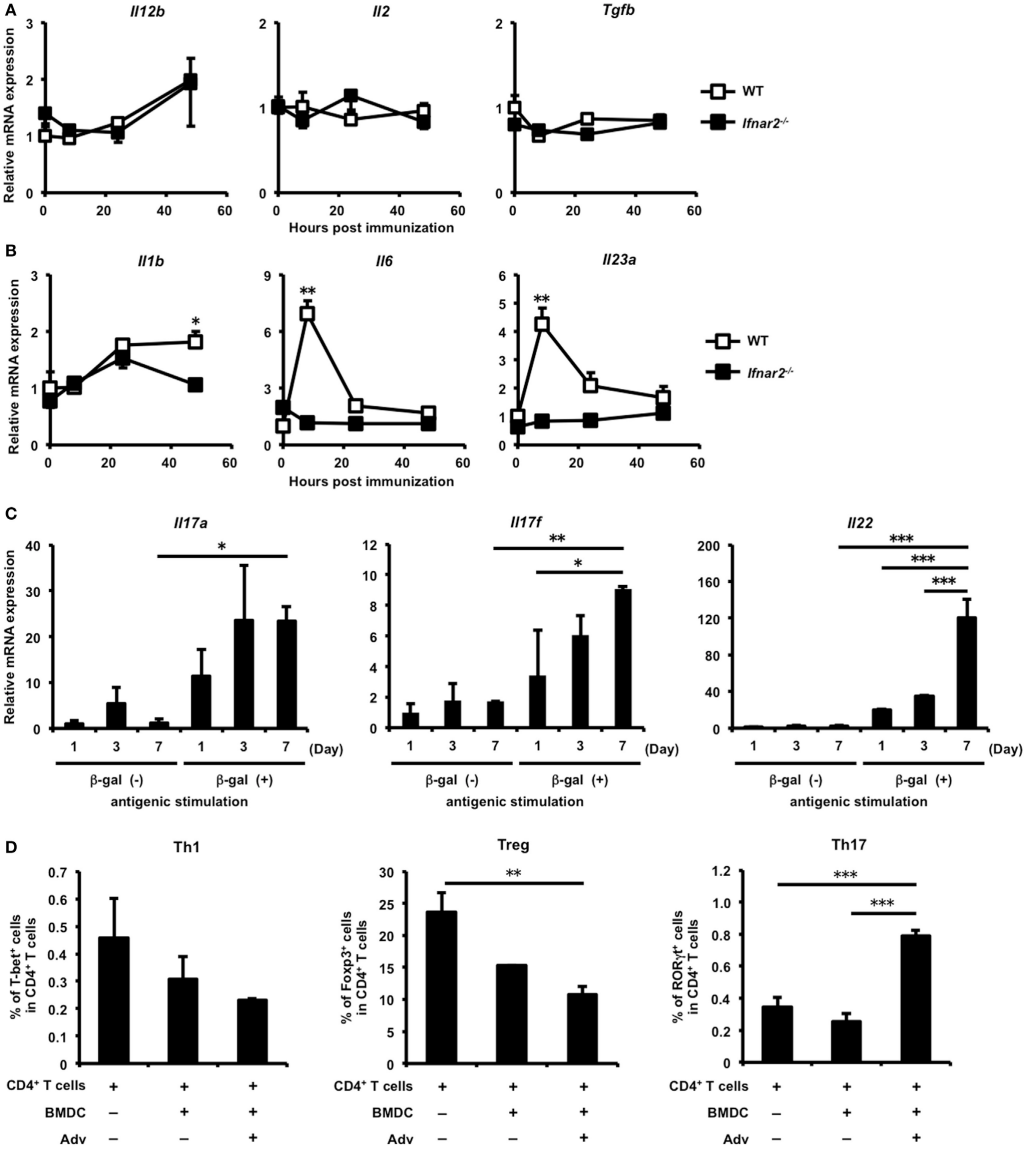

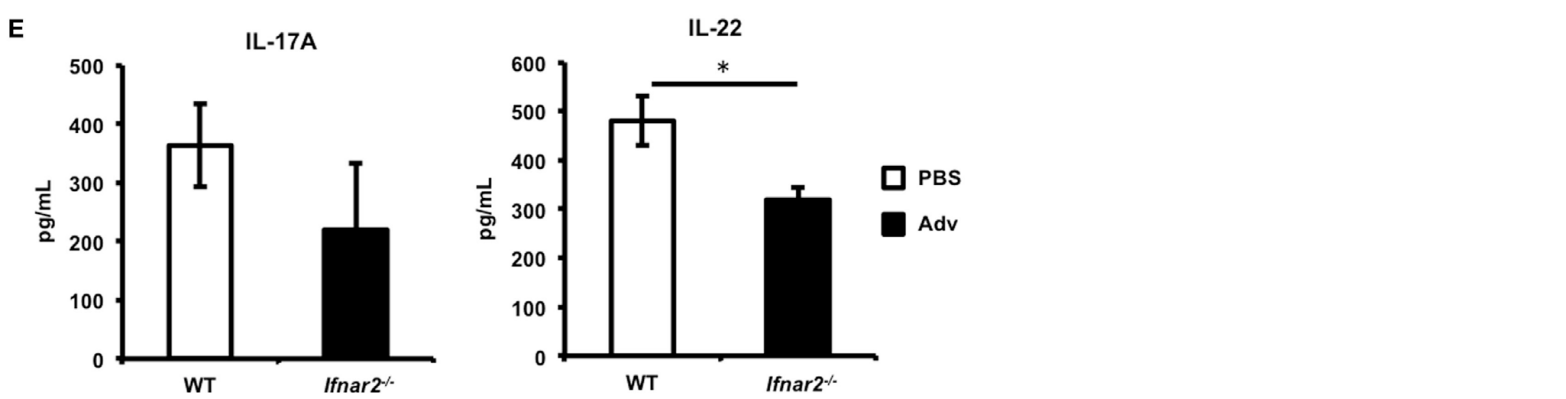
Induction of antigen-specific T helper 17 cells in the inguinal lymph nodes (iLNs) *via* type I interferon signaling following intramuscular (*i.m*.) adenovirus vector (Adv) vaccination. **(A,B)** At 0, 8, 24, and 48 h after *i.m*. Ad-LacZ vaccination, total RNA was extracted from mononuclear cells in iLNs, and mRNA expression of **(A)** *Il12b, Il2*, tumor growth factor *b*, **(B)** *Il1b, Il6*, and *Il23a* was measured by quantitative reverse transcription-polymerase chain reaction (qRT-PCR) and normalized against *Actb* expression. Graphs represent the relative mRNA expression of each gene normalized against its expression in wild-type (WT) mice at 0 h. **(C)** At 1, 3, and 7 days after *i.m*. Ad-LacZ vaccination, CD4^+^ T cells in the iLNs of WT mice were sorted and co-cultured with splenic dendritic cells (DCs) in the presence of β-gal for 24 h. The mRNA expression of *Il17a, Il17f*, and *Il22* was measured by qRT-PCR and normalized against *Actb* expression. Graphs represent the relative mRNA expression of each gene normalized against its level in non-stimulated CD4^+^ T cells on day 1. **(D)** Enriched CD4^+^ T cells from spleens were co-cultured with Adv-infected bone-marrow-derived DCs, and the frequencies of T-bet^+^, GATA-3^+^, Foxp3^+^, and RORγt^+^ cells in CD4^+^ T cell populations were measured by flow cytometry. **(E)** At 7 days after vaccination, CD4^+^ T cells in iLNs were sorted and co-cultured with splenic DCs in the presence of β-gal for 4 days. The amounts of IL-17A and IL-22 were measured by enzyme-linked immunosorbent assay. Data are **(A,B)** the pools of three independent experiments and are shown as the mean ± SEM (*n* = 3). Data are representative of **(C,E)** two and **(D)** three independent experiments and are shown as the mean ± SD (*n* = 3). *p < 0.05; **p < 0.01; ***p < 0.001 [**(A,B)** Student’s *t*-test at each time point; **(C,D)** one-way analysis of variance; **(E)** Student’s *t*-test].

T helper cells play important roles in CTL induction (33–35). We hypothesized that type I IFN signaling regulates Th-cell induction, resulting in the subsequent induction of gut-mucosal CTLs. To investigate whether type I IFN signaling affects Th-cell induction, we first checked the expression of several cytokines related to differentiation into each Th-cell subset. The expression of interleukin (*Il*)*12b*, which is related to the differentiation of Th1 cells, and *Il2* and tumor growth factor (*Tgf)b*, which are related to the differentiation of regulatory T (Treg) cells, were similar in WT and *Ifnar2^−/−^* mice (Figure 2A). By contrast, the expression of the Th17-polarizing cytokines *Il6, Il1b*, and *Il23a* was up-regulated in the iLNs of WT mice, but not in those of *Ifnar2^−/−^* mice (Figure 2B). Additionally, the expression of *Tgfb*, which is also involved in Th17 differentiation (36), was not up-regulated in WT mice. Because recent studies reported that Th17 cells differentiate in the presence of IL-6, IL-1β, and IL-23 (37, 38), we expected that Th17 cells would differentiate in the iLNs of WT mice following *i.m*. Adv vaccination. Therefore, these data suggest that Th17 differentiation, but not that of Th1 or Treg cells, was preferentially induced in iLNs through type I IFN signaling following *i.m*. Adv vaccination.

### Th17 Differentiation Is Induced in iLNs through Type I IFN Signaling

To examine the induction of Ag-specific Th17 cells in iLNs, CD4^+^ T cells purified from the iLNs of Adv-administered mice were co-cultured with Ag-presenting cells in the presence of β-gal, followed by measurement of Th17-producing cytokines. In iLN CD4^+^ T cells from Adv-administered WT mice, the mRNA expression of the Th17-producing cytokines *Il17a, Il17f*, and *Il22* was significantly up-regulated following *in vitro* Ag-specific stimulation (Figure 2C), indicating that Ag-specific Th17 cells were induced in the iLNs of WT mice following *i.m*. Adv vaccination. Consistent with these data, we observed that Adv-infected BMDCs induced Th17 differentiation (Figure 2D). Moreover, after 7 days, IL-17A levels in iLN CD4^+^ T cells were 363 ± 70.5 pg/mL (for WT mice) and 220 ± 112 pg/mL (for *Ifnar2^−/−^* mice), with no significant difference between them. In addition, IL-22 level in iLN CD4^+^ T cells from *Ifnar2^−/−^* mice was significantly lower than the level in iLN CD4^+^ T cells from WT mice (Figure 2E). These data suggest that type I IFN signaling is important for the induction of Ag-specific Th17 cells in iLNs following Adv vaccination.

### Type I IFN Signaling Initiates Recruitment of Inflammatory DCs (inf DCs) to iLNs and Their Subsequent Activation

**Figure 3:**
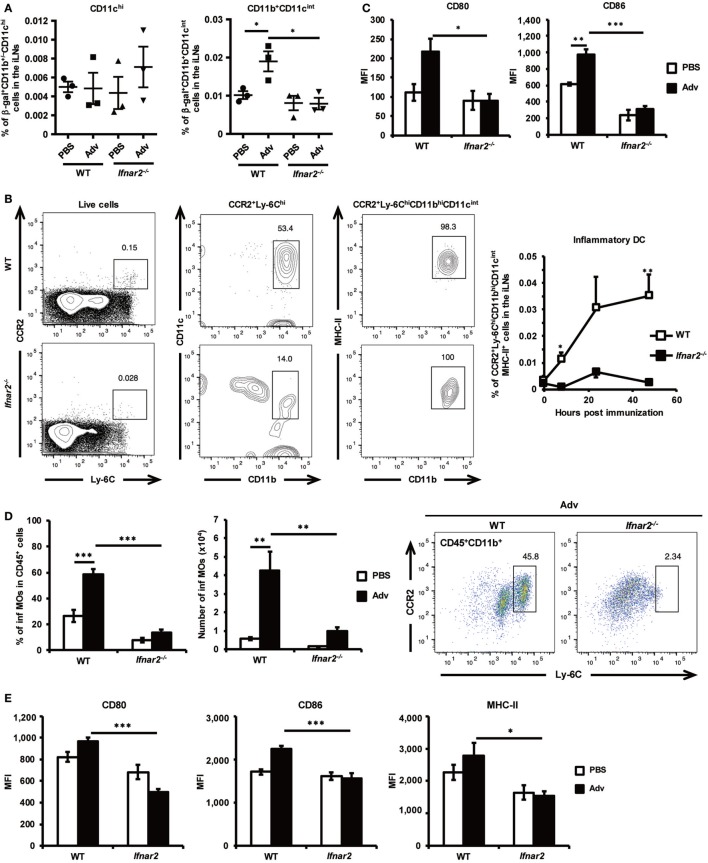

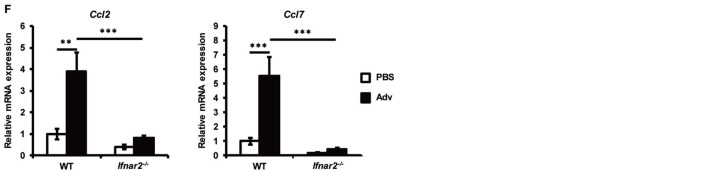
Inflammatory dendritic cells (inf DCs) are recruited to inguinal lymph nodes (iLNs) through type I interferon signaling. **(A)** At 8 h after intramuscular Ad-LacZ vaccination, the frequency of β-gal^+^ cells in iLNs was measured by flow cytometry. **(B)** Inf DCs were gated as CCR2^+^Ly-6C^hi^MHC-II^+^CD11b^hi^CD11c^int^ cells. At 0, 8, 24, and 48 h after vaccination, the frequency of inf DCs in iLNs was measured by flow cytometry. **(C)** At 48 h after vaccination, the expression of the activation markers CD80 and CD86 on inf DCs in iLNs was analyzed by flow cytometry. **(D)** At 24 h after vaccination, the frequency of CCR2^+^Ly-6C^hi^CD11b^+^ inflammatory monocytes (inf MOs) in CD45^+^ cells from quadriceps muscles was measured by flow cytometry. **(E)** At 48 h after vaccination, the expression of the activation markers CD80, CD86, and MHC-II on inf MOs from quadriceps muscles was analyzed by flow cytometry. **(F)** At 8 h after vaccination, mRNA expression of *Ccl2* and *Ccl7* was measured by quantitative reverse transcription-polymerase chain reaction and normalized against *Gapdh* expression. Graphs represent the relative mRNA expression of each gene normalized against its expression in PBS-administrated wild-type mice. Data are the pools of **(A,C–F)** three and **(B)** four independent experiments and are shown as the mean ± SEM [**(A)**: *n* = 3; **(B)**: *n* = 4–5; **(C–E)**: *n* = 5–6; **(F)**: *n* = 8–9]. *p < 0.05; **p < 0.01; ***p < 0.001 [**(A, C–F)**: one-way analysis of variance; **(B)** Student’s *t*-test at each time point].

We considered the possibility that the disruption of the *Ifnar2* gene in CD4^+^ T cells might affect Th17 differentiation in iLNs. However, we confirmed that *Ifnar2^−/−^* CD4^+^ T cells differentiated normally into Th17 cells in the presence of IL-6, IL-1β, and IL-23 *in vitro* (Figure S1 in Supplementary Material). Both polarizing cytokines and myeloid cells, which are responsible for presenting Ag to T cells, are essential for the induction of Th-cell differentiation. To determine the types of myeloid cells involved in Th17 differentiation in iLNs, we analyzed β-gal-bearing myeloid-cell populations in iLNs at 8 h after Adv vaccination according to flow cytometry-based detection of β-gal^+^ cells using a fluorogenic galactosidase substrate (39). In WT mice, although the frequency of β-gal^+^CD11b^+/−^CD11c^hi^ cells failed to increase at this early time point following *i.m*. Adv vaccination, we observed elevations in the frequency of β-gal^+^CD11b^+^CD11c^int^ cells (Figure 3A). However, the amount of β-gal^+^CD11b^+^CD11c^int^ cells in the iLNs of *Ifnar2^−/−^* mice was significantly lower than the levels in WT mice, suggesting that Ag-bearing CD11b^+^CD11c^int^ cells were recruited to iLNs *via* type I IFN signaling. Previous studies reported that inf DCs, which are included in CD11b^+^CD11c^int^ populations, induce Th17 differentiation under inflammatory conditions in humans and mice (40, 41). As expected, we found that the frequency of inf DCs in the iLNs of WT mice increased following Adv vaccination (Figure 3B; Figure S2A in Supplementary Material). By contrast, the inf DC frequency in *Ifnar2^−/−^* mice did not increase, indicating that inf DCs were recruited to iLNs *via* type I IFN signaling. Additionally, we observed lower expression of the activation markers CD80 and CD86 on inf DCs in *Ifnar2^−/−^* mice than that in WT mice (Figure 3C). These results suggest that inf DCs were activated through type I IFN signaling and were capable of inducing Th17 differentiation in iLNs. In general, inf DCs differentiate from inflammatory monocytes (inf MOs), and during infection and inflammation, inf MOs are recruited to inflamed tissue and activated by innate immune signaling (42). We speculated that inf MOs were recruited to the quadriceps muscles, which were the vaccination sites in this study. Because the mechanisms associated with activation of the innate immune response at muscle sites following *i.m*. Adv vaccination have not been clarified, we investigated this activity in mouse quadriceps muscles. At 24 h after Adv vaccination, we observed that the frequency of inf MOs, but not neutrophils or macrophages, significantly increased in the muscles of WT mice (Figure 3D; Figure S2B in Supplementary Material). However, in Adv-administered *Ifnar2^−/−^* mice, the frequency of inf MOs did not increase, indicating that inf MOs were preferentially recruited to the muscles by type I IFN signaling. Moreover, we observed significantly lower expression of activation markers on inf MOs in *Ifnar2^−/−^* mice when compared with levels observed in WT mice (Figure 3E). Multiple reports showed that CCR2 ligands, such as C–C chemokine ligand (CCL)2 and CCL7, mediate inf MO recruitment under inflammatory conditions (42, 43). We hypothesized that type I IFN-signaling-dependent recruitment of inf MOs was regulated by these chemokines, subsequently finding that the expression of CCL2 and CCL7 was up-regulated in the muscles of WT mice, but not in those of *Ifnar2^−/−^* mice (Figure 3F). These data indicate that type I IFN signaling initiated recruitment of inf MOs to muscles through induction of CCL2 and CCL7 expression. Our findings suggest that the induction of inf DCs in iLNs was dependent on type I IFN-signaling-related trafficking of inf MOs to the muscles.

### Reduction of Th17 Cells in the Gut Mucosa of *Ifnar2^−/−^* Mice

**Figure 4:**
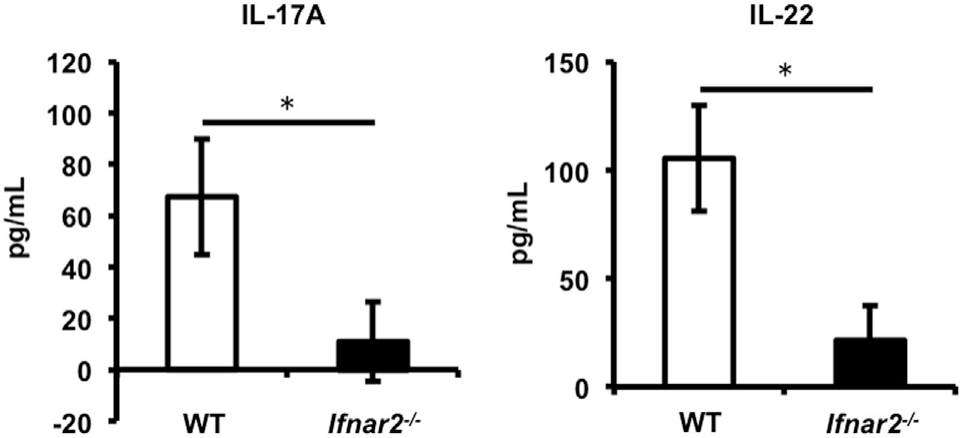
Type I interferon signaling is required for induction of antigen-specific T helper 17 cells in the gut mucosa following intramuscular adenovirus vector vaccination. At 2 weeks after vaccination, CD4^+^ T cells in the lamina propria were sorted and co-cultured with splenic dendritic cells in the presence of β-gal for 4 days, and the amounts of IL-17A and IL-22 were measured by enzyme-linked immunosorbent assay. Data are representative of three independent experiments and are shown as the mean ± SD (*n* = 3). *p < 0.05 (Student’s *t*-test).

We demonstrated that type I IFN signaling is important for induction of Th17 cells in iLNs. Consistent with previous studies (37, 38, 44), our results shown in Figures 2A,B also suggest that naïve CD4^+^ T cells differentiate into pro-inflammatory Th17 cells in the presence of IL-1β. Recent studies have reported that Th17 cells promote CTL proliferation and activation in tumor-bearing mice (45–47), and in some of these reports, Th17 cells that differentiated in the presence of IL-1β were also capable of promoting CTL induction to a greater extent than those in the presence of TGF-β (46, 47). Furthermore, Th17 cells also exhibit gut-homing capacity (48–50). Taken together, these results led us to hypothesize that Th17 cells induced in iLNs after Adv vaccination would be able to migrate to the gut mucosa and to promote the induction of Ag-specific gut-mucosal CTLs. To test this hypothesis, we assessed the induction of Ag-specific gut-mucosal Th17 cells. Determination of the production of the Th17-producing cytokines IL-17A and IL-22 following Ag stimulation of gut-mucosal CD4^+^ T cells in *Ifnar2^−/−^* mice revealed significantly lower levels when compared with those in WT mice (Figure 4). These data indicate that type I IFN signaling was required for induction of Ag-specific Th17 cells in the gut mucosa.

### Th17 Cells Promote the Induction of Ag-Specific CTLs in Gut Mucosa

**Figure 5:**
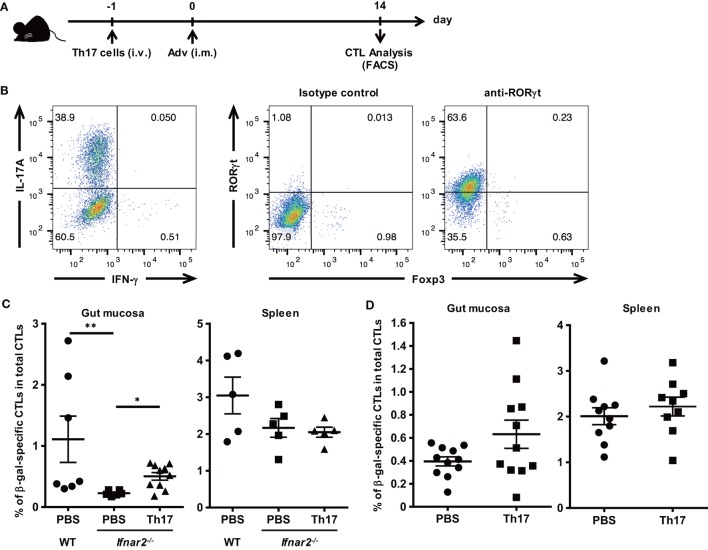
T helper 17 (Th17) cells promote the induction of antigen-specific gut-mucosal cytotoxic T lymphocytes. **(A)** *In vitro*-differentiated Th17 cells were administered through the tail vein at 1 day prior to intramuscular Ad-LacZ vaccination. At 2 weeks after vaccination, the gut mucosa and spleens were collected. **(B)** Naïve CD4^+^ T cells were cultured under Th17 differentiation conditions for 4 days, and the frequency of IL-17A^+^ and RORγt^+^ cells was determined using flow cytometry. **(C,D)** The frequency of β-gal-specific CD8^+^ T cells in the CD8^+^ T cell population in tissues of wild-type (WT) mice, **(C)** *Ifnar2^−/−^* mice, Th17-transferred *Ifnar2^−/−^* mice, and **(D)** Th17-transferred WT mice was measured by flow cytometry. Data are the pools of **(C)** three and **(D)** four independent experiments and are shown as the means ± SEM [**(C)**: spleen (PBS, Th17; *n* = 5); gut mucosa (PBS; *n* = 6–7, Th17; *n* = 10); **(D)**: *n* = 9–11]. *p < 0.05; **p < 0.01 [**(C)** Kruskal–Wallis test; **(D)** Student’s *t*-test].

To determine whether Th17 cells promote the induction of Ag-specific CTLs in the gut mucosa of *Ifnar2^−/−^* mice, we transferred *in vitro*-differentiated Th17 cells into *Ifnar2^−/−^* mice prior to Adv vaccination (Figures 5A,B). At 2 weeks after Adv vaccination, the frequency of β-gal-specific CTLs in the spleens of *Ifnar2^−/−^* mice did not increase after Th17 transfer (Figure 5C). By contrast, we observed increases in β-gal-specific CTLs in the gut mucosa of *Ifnar2^−/−^* mice following Th17 transfer to the level of WT mice (Figure 5C), indicating that Th17 cells played an important role in the induction of Ag-specific CTLs in the gut mucosa through type I IFN signaling. To test the hypothesis that enhancement of Th17 induction could promote the induction of gut-mucosal CTLs, we transferred *in vitro*-differentiated Th17 cells into WT mice (Figure 5D). As expected, while the frequency of occurrence of β-gal-specific CTLs in the spleen increased by 1.10-folds after Th17 transfer, that in the gut mucosa increased by 1.60-folds. These data indicate that Th17 cells promote the induction of Ag-specific CTLs in the gut-mucosal compartment, but not in systemic compartments.

## Discussion

Although Adv is among the most promising vectors for vaccine development due to its ability to establish functional and durable adaptive immunity in both systemic and gut-mucosal compartments, the molecular mechanisms associated with how Adv induces gut-mucosal immunity remain to be clarified. In a previous study, we showed that type I IFN signaling following *i.m*. Adv vaccination was required for induction of Ag-specific CTLs in the gut-mucosal site, but not in the systemic site (24). Type I IFNs suppress transgene expression (51, 52), with Quinn et al. showing that excessive type I IFN expression initiated by polyI:C injection limits Ag expression and systemic adaptive immunity following *i.m*. Adv vaccination (53). These data suggest that enhancement of adaptive mucosal immunity would not be promoted by induction of excessive type I IFN production. Therefore, to investigate mechanisms for strengthening adaptive immunity in gut mucosa by *i.m*. Adv vaccination, we explored the physiological role of type I IFN signaling in Adv-vaccination-induced Ag-specific CTLs in gut mucosa. Our findings revealed cell types and molecules regulated by type I IFN signaling that might constitute viable targets for improving Adv-vaccine efficacy.

We found type I IFN signaling dispensable for induction of gut-homing Ag-specific CTLs and indispensable for induction of Ag-specific Th17 cells in iLNs and gut mucosa following *i.m*. Adv vaccination. Th17 cells are associated with clearance of some microorganisms and cancers, as well as the pathogenesis of autoimmune and inflammatory diseases (54, 55). However, the relationship between Th17 cells and a CTL-inducing vaccine, such as Adv, remains unclear. Surprisingly, we found that Th17 cells promoted induction of Ag-specific CTLs in the gut-mucosal compartment, but not in the systemic compartment, following *i.m*. Adv vaccination. Because Th17 cells enhanced the induction of gut-mucosal CTLs, we hypothesized that cytokines produced from Th17 cells are important in this process. IL-17 targets non-immune cells, resulting in the production of several pro-inflammatory cytokines and chemokines, including tumor necrosis factor-α, granulocyte-macrophage colony stimulating factor, prostaglandin E2, CCL2, and CCL20 (54, 55). Therefore, Th17 cells might migrate to the gut mucosa and, through their IL-17 production, activate residential stromal cells to secrete other chemokines, resulting in CTL recruitment to the gut mucosa. Additionally, IL-21, also produced by Th17 cells (54, 55), might promote the proliferation of tumor Ag-specific CTLs (56–58). Therefore, it is also possible that IL-21 might influence the induction of Ag-specific CTLs in gut mucosa. In future experiments, the mechanisms associated with vaccine-mediated Th17-cell induction of Ag-specific CTLs will be explored.

Here, we demonstrated that Ag-specific CTLs and Th17 cells were induced in the gut mucosa following *i.m*. Adv vaccination. Although CTLs establish immunological protection against intracellular pathogens, recent studies showed that Th17 cells play a key role in the induction of protective immunity against extracellular pathogens in the gut mucosa, with Th17 responses initiating production of antimicrobial peptides by epithelial cells in the intestinal lumen and the recruitment of monocytes, macrophages, and neutrophils to gut-mucosal sites (59–61). Additionally, IL-17A secretion is capable of enhancing phagocytosis of pathogens by neutrophils (62). Therefore, our findings are important because they suggest that the Adv vaccine is capable of inducing both CTL and Th17 responses to initiate the clearance of various intracellular and extracellular pathogens.

**Figure 6:**
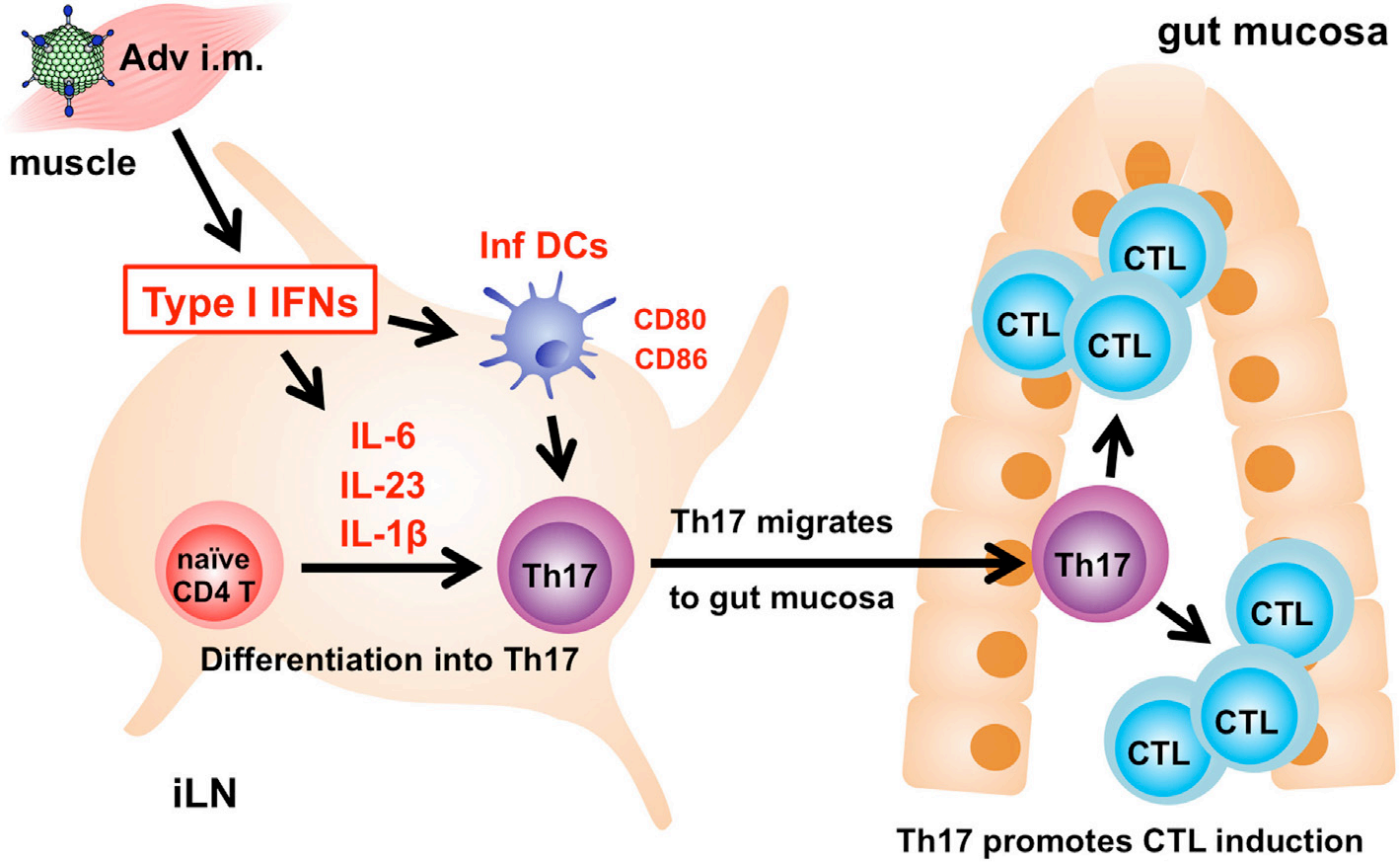
The mechanism associated with induction of antigen (Ag)-specific gut-mucosal cytotoxic T lymphocytes (CTLs) *via* type I interferon (IFN) signaling. Following intramuscular adenovirus vector vaccination, type I IFN signaling induces Ag-specific T helper 17 (Th17) cells in inguinal lymph nodes (iLNs), followed by Th17-cell migration from iLNs to the gut mucosa to promote the induction of Ag-specific CTLs.

In summary, we revealed the molecular mechanism associated with induction of gut-mucosal CTLs through type I IFN signaling following *i.m*. Adv vaccination (Figure 6). Our results show that Th17 cells translate systemic type I IFN signaling into a gut-mucosal CTL response following Adv vaccination, and that the enhancement of Th17 induction promotes CTL responses. To the best of our knowledge, this study is the first to suggest that Th17 cells induce Ag-specific CTLs exclusively in the gut mucosa. These findings promote the development of safer and more efficient Adv vaccines capable of establishing protective and durable immunity in both systemic and gut-mucosal compartments.

## Ethics Statement

All animal-based experimental procedures used in this study were performed in accordance with the institutional guidelines for animal experiments at Osaka University (Douyaku 28-3-1) and the National Institutes of Biomedical Innovation, Health and Nutrition (DS19-106).

## Author Contributions

MH designed and performed the experiments, analyzed the data, and wrote the manuscript. MT supervised the experiments, provided technical support and critical discussions related to the manuscript, and wrote the manuscript. NF performed some of the experiments and analyzed the data. KK, KI, and SA provided the *Ifnar2^−/−^* mice and critical reviews of the manuscript. MS provided critical reviews of the manuscript. FS provided critical discussions related to the manuscript. HM supervised the experiments and wrote the manuscript. All of the authors have reviewed the manuscript.

## Conflict of Interest Statement

The authors declare that the research was conducted in the absence of any commercial or financial relationships that could be construed as a potential conflict of interest.

## References

[1] Holmgren J Czerkinsky C. Mucosal immunity and vaccines. Nat Med (2005) 11:S45–53. 10.1038/nm1213 15812489

[2] Belyakov IM Ahlers JD. Functional CD8+ CTLs in mucosal sites and HIV infection: moving forward toward a mucosal AIDS vaccine. Trends Immunol (2008) 29:574–85. 10.1016/j.it.2008.07.010 18838298

[3] Neutra MR Kozlowski PA. Mucosal vaccines: the promise and the challenge. Nat Rev Immunol (2006) 6:148–58. 10.1038/nri1777 16491139

[4] Tatsis N Ertl HCJ. Adenoviruses as vaccine vectors. Mol Ther (2004) 10:616–29. 10.1016/j.ymthe.2004.07.013 15451446 PMC7106330

[5] Tatsis N Lasaro MO Lin S-W Haut LH Xiang ZQ Zhou D Adenovirus vector-induced immune responses in nonhuman primates: responses to prime boost regimens. J Immunol (2009) 182:6587–99. 10.4049/jimmunol.0900317 19414814 PMC2711537

[6] Stanley DA Honko AN Asiedu C Trefry JC Lau-Kilby AW Johnson JC Chimpanzee adenovirus vaccine generates acute and durable protective immunity against ebolavirus challenge. Nat Med (2014) 20:1126–9. 10.1038/nm.3702 25194571

[7] Barnes E Folgori A Capone S Swadling L Aston S Kurioka A Novel adenovirus-based vaccines induce broad and sustained T cell responses to HCV in man. Sci Transl Med (2012) 4:115ra1. 10.1126/scitranslmed.3003155 22218690 PMC3627207

[8] Kaufman DR Liu J Carville A Mansfield KG Havenga MJE Goudsmit J Trafficking of antigen-specific CD8+ T lymphocytes to mucosal surfaces following intramuscular vaccination. J Immunol (2008) 181:4188–98. 10.4049/jimmunol.181.6.4188 18768876 PMC2580672

[9] Kaufman DR Bivas-Benita M Simmons NL Miller D Barouch DH. Route of adenovirus-based HIV-1 vaccine delivery impacts the phenotype and trafficking of vaccine-elicited CD8+ T lymphocytes. J Virol (2010) 84:5986–96. 10.1128/JVI.02563-09 20357087 PMC2876628

[10] Liu J O’Brien KL Lynch DM Simmons NL La Porte A Riggs AM Immune control of an SIV challenge by a T-cell-based vaccine in rhesus monkeys. Nature (2009) 457:87–91. 10.1038/nature07469 18997770 PMC2614452

[11] Kawai T Akira S. Toll-like receptors and their crosstalk with other innate receptors in infection and immunity. Immunity (2011) 34:637–50. 10.1016/j.immuni.2011.05.006 21616434

[12] Takeuchi O Akira S Pattern recognition receptors and inflammation. Cell (2010) 140:805–20. 10.1016/j.cell.2010.01.022 20303872

[13] Kawai T Akira S Innate immune recognition of viral infection. Nat Immunol (2006) 7:131–7. 10.1038/ni1303 16424890

[14] Pulendran B Ahmed R. Translating innate immunity into immunological memory: implications for vaccine development. Cell (2006) 124:849–63. 10.1016/j.cell.2006.02.019 16497593

[15] Pulendran B Ahmed R. Immunological mechanisms of vaccination. Nat Immunol (2011) 12:509–17. 10.1038/ni.2039 21739679 PMC3253344

[16] Hartman ZC Kiang A Everett RS Serra D Yang XY Clay TM Adenovirus infection triggers a rapid, MyD88-regulated transcriptome response critical to acute-phase and adaptive immune responses in vivo. J Virol (2007) 81:1796–812. 10.1128/JVI.01936-06 17121790 PMC1797572

[17] Zhu J Huang X Yang Y. Innate immune response to adenoviral vectors is mediated by both toll-like receptor-dependent and -independent pathways. J Virol (2007) 81:3170–80. 10.1128/JVI.02192-06 17229689 PMC1866082

[18] Yamaguchi T Kawabata K Koizumi N Sakurai F Nakashima K Sakurai H Role of MyD88 and TLR9 in the innate immune response elicited by serotype 5 adenoviral vectors. Hum Gene Ther (2007) 18:753–62. 10.1089/hum.2007.016 17685831

[19] Lam E Stein S Falck-Pedersen E Adenovirus detection by the cGAS/STING/TBK1 DNA sensing cascade. J Virol (2013) 88:974–81. 10.1128/JVI.02702-13 24198409 PMC3911663

[20] Yamaguchi T Kawabata K Kouyama E Ishii KJ Katayama K Suzuki T Induction of type I interferon by adenovirus-encoded small RNAs. Proc Natl Acad Sci U S A (2010) 107:17286–91. 10.1073/pnas.1009823107 20855616 PMC2951419

[21] Hensley SE Giles-Davis W McCoy KC Weninger W Ertl HCJ Dendritic cell maturation, but not CD8+ T cell induction, is dependent on type I IFN signaling during vaccination with adenovirus vectors. J Immunol (2005) 175:6032–41. 10.4049/jimmunol.175.9.6032 16237098

[22] Mueller SN Germain RN. Stromal cell contributions to the homeostasis and functionality of the immune system. Nat Rev Immunol (2009) 9:618–29. 10.1038/nri2588 19644499 PMC2785037

[23] Zhu J Huang X Yang Y. Type I IFN signaling on both B and CD4 T cells is required for protective antibody response to adenovirus. J Immunol (2007) 178:3505–10. 10.4049/jimmunol.178.6.3505 17339445

[24] Shoji M Tachibana M Katayama K Tomita K Tsuzuki S Sakurai F Type-I IFN signaling is required for the induction of antigen-specific CD8(+) T cell responses by adenovirus vector vaccine in the gut-mucosa. Biochem Biophys Res Commun (2012) 425:89–93. 10.1016/j.bbrc.2012.07.056 22819843

[25] Ishii KJ Kawagoe T Koyama S Matsui K Kumar H Kawai T TANK-binding kinase-1 delineates innate and adaptive immune responses to DNA vaccines. Nature (2008) 451:725–9. 10.1038/nature06537 18256672

[26] Kawabata K Sakurai F Yamaguchi T Hayakawa T Mizuguchi H. Efficient gene transfer into mouse embryonic stem cells with adenovirus vectors. Mol Ther (2005) 12:547–54. 10.1016/j.ymthe.2005.04.015 15950541

[27] Hemmi M Tachibana M Tsuzuki S Shoji M Sakurai F Kawabata K The early activation of CD8+ T cells is dependent on type I IFN signaling following intramuscular vaccination of adenovirus vector. Biomed Res Int (2014) 2014:1–6. 10.1155/2014/158128 24971314 PMC4058243

[28] Niwa H Yamamura K Miyazaki J. Efficient selection for high-expression transfectants with a novel eukaryotic vector. Gene (1991) 108:193–9. 10.1016/0378-1119(91)90434-D 1660837

[29] Maizel JV White DO Scharff MD The polypeptides of adenovirus. I. Evidence for multiple protein components in the virion and a comparison of types 2, 7A, and 12. Virology (1968) 36:115–25. 10.1016/0042-6822(68)90121-9 5669982

[30] Shoji M Katayama K Tachibana M Tomita K Sakurai F Kawabata K Intramuscular DNA immunization with in vivo electroporation induces antigen-specific cellular and humoral immune responses in both systemic and gut-mucosal compartments. Vaccine (2012) 30:7278–85. 10.1016/j.vaccine.2012.09.046 23036499

[31] Tsuzuki S Tachibana M Hemmi M Yamaguchi T Shoji M Sakurai F TANK-binding kinase 1-dependent or -independent signaling elicits the cell-type-specific innate immune responses induced by the adenovirus vector. Int Immunol (2016) 28:105–15. 10.1093/intimm/dxv058 26489883

[32] Ganguly S Manicassamy S Blackwell J Pulendran B Amara RR. Adenovirus type 5 induces vitamin A-metabolizing enzymes in dendritic cells and enhances priming of gut-homing CD8 T cells. Mucosal Immunol (2011) 4:528–38. 10.1038/mi.2011.1 21289616 PMC3097311

[33] Shedlock DJ Shen H. Requirement for CD4 T cell help in generating functional CD8 T cell memory. Science (2003) 300:337–9. 10.1126/science.1082305 12690201

[34] Yang TC Millar J Groves T Zhou W Grinshtein N Parsons R On the role of CD4+ T cells in the CD8+ T-cell response elicited by recombinant adenovirus vaccines. Mol Ther (2007) 15:997–1006. 10.1038/sj.mt.6300130 17375073

[35] Provine NM Larocca RA Penaloza-Macmaster P Borducchi EN McNally A Parenteau LR Longitudinal requirement for CD4+ T cell help for adenovirus vector-elicited CD8+ T cell responses. J Immunol (2014) 192(11):5214–25. 10.4049/jimmunol.1302806 24778441 PMC4025612

[36] Mangan PR Harrington LE O’Quinn DB Helms WS Bullard DC Elson CO Transforming growth factor-beta induces development of the T(H)17 lineage. Nature (2006) 441:231–4. 10.1038/nature04754 16648837

[37] Ghoreschi K Laurence A Yang X-P Tato CM McGeachy MJ Konkel JE Generation of pathogenic T(H)17 cells in the absence of TGF-β signalling. Nature (2010) 467:967–71. 10.1038/nature09447 20962846 PMC3108066

[38] Chung Y Chang SH Martinez GJ Yang XO Nurieva R Kang HS Critical regulation of early Th17 cell differentiation by interleukin-1 signaling. Immunity (2009) 30:576–87. 10.1016/j.immuni.2009.02.007 19362022 PMC2705871

[39] Wei G Hong W Detection of LacZ expression by FACS-Gal analysis. Protoc Exch (2008). 10.1038/nprot.2008.163

[40] Segura E Touzot M Bohineust A Cappuccio A Chiocchia G Hosmalin A Human inflammatory dendritic cells induce Th17 cell differentiation. Immunity (2013) 38:336–48. 10.1016/j.immuni.2012.10.018 23352235

[41] Ko H-J Brady JL Ryg-Cornejo V Hansen DS Vremec D Shortman K GM-CSF-responsive monocyte-derived dendritic cells are pivotal in Th17 pathogenesis. J Immunol (2014) 192:2202–9. 10.4049/jimmunol.1302040 24489100

[42] Shi C Pamer EG. Monocyte recruitment during infection and inflammation. Nat Rev Immunol (2011) 11:762–74. 10.1038/nri3070 21984070 PMC3947780

[43] Tsou C-L Peters W Si Y Slaymaker S Aslanian AM Weisberg SP Critical roles for CCR2 and MCP-3 in monocyte mobilization from bone marrow and recruitment to inflammatory sites. J Clin Invest (2007) 117:902–9. 10.1172/JCI29919 17364026 PMC1810572

[44] Zielinski CE Mele F Aschenbrenner D Jarrossay D Ronchi F Gattorno M Pathogen-induced human TH17 cells produce IFN-γ or IL-10 and are regulated by IL-1β. Nature (2012) 484:514–8. 10.1038/nature10957 22466287

[45] Martin-Orozco N Muranski P Chung Y Yang XO Yamazaki T Lu S T helper 17 cells promote cytotoxic T cell activation in tumor immunity. Immunity (2009) 31:787–98. 10.1016/j.immuni.2009.09.014 19879162 PMC2787786

[46] Chalmin F Mignot G Bruchard M Chevriaux A Végran F Hichami A Stat3 and Gfi-1 transcription factors control Th17 cell immunosuppressive activity via the regulation of ectonucleotidase expression. Immunity (2012) 36:362–73. 10.1016/j.immuni.2011.12.019 22406269

[47] Chatterjee S Thyagarajan K Kesarwani P Song JH Soloshchenko M Fu J Reducing CD73 expression by IL1β-programmed Th17 cells improves immunotherapeutic control of tumors. Cancer Res (2014) 74:6048–59. 10.1158/0008-5472.CAN-14-1450 25205101 PMC4216762

[48] Wang C Kang SG Lee J Sun Z Kim CH. The roles of CCR6 in migration of Th17 cells and regulation of effector T-cell balance in the gut. Mucosal Immunol (2009) 2:173–83. 10.1038/mi.2008.84 19129757 PMC2709747

[49] Wang C Kang SG HogenEsch H Love PE Kim CH. Retinoic acid determines the precise tissue tropism of inflammatory Th17 cells in the intestine. J Immunol (2010) 184:5519–26. 10.4049/jimmunol.0903942 20400707 PMC3009589

[50] Hirota K Turner J-E Villa M Duarte JH Demengeot J Steinmetz OM Plasticity of TH17 cells in Peyer’s patches is responsible for the induction of T cell-dependent IgA responses. Nat Immunol (2013) 14:372–9. 10.1038/ni.2552 23475182 PMC3672955

[51] Christ M Lusky M Stoeckel F Dreyer D Dieterlé A Michou AI Gene therapy with recombinant adenovirus vectors: evaluation of the host immune response. Immunol Lett (1997) 57:19–25. 10.1016/S0165-2478(97)00049-7 9232420

[52] Acsadi G O’Hagan D Lochmüller H Prescott S Larochelle N Nalbantoglu J Interferons impair early transgene expression by adenovirus-mediated gene transfer in muscle cells. J Mol Med (Berl) (1998) 76:442–50. 10.1007/s001090050236 9625301

[53] Quinn KM Zak DE Costa A Yamamoto A Kastenmuller K Hill BJ Antigen expression determines adenoviral vaccine potency independent of IFN and STING signaling. J Clin Invest (2015) 125:1129–46. 10.1172/JCI78280 25642773 PMC4362254

[54] Korn T Bettelli E Oukka M Kuchroo VK IL-17 and Th17 cells. Annu Rev Immunol (2009) 27:485–517. 10.1146/annurev.immunol.021908.132710 19132915

[55] Maddur MS Miossec P Kaveri SV Bayry J Th17 cells. Am J Pathol (2012) 181:8–18. 10.1016/j.ajpath.2012.03.044 22640807

[56] Zeng R Spolski R Finkelstein SE Oh S Kovanen PE Hinrichs CS Synergy of IL-21 and IL-15 in regulating CD8^+^ T cell expansion and function. J Exp Med (2005) 201:139–48. 10.1084/jem.20041057 15630141 PMC2212766

[57] Li Y Bleakley M Yee C. IL-21 influences the frequency, phenotype, and affinity of the antigen-specific CD8 T cell response. J Immunol (2005) 175:2261–9. 10.4049/jimmunol.175.4.2261 16081794

[58] Sutherland APR Joller N Michaud M Liu SM Kuchroo VK Grusby MJ. IL-21 promotes CD8+ CTL activity via the transcription factor T-bet. J Immunol (2013) 190:3977–84. 10.4049/jimmunol.1201730 23479229

[59] Aujla SJ Dubin PJ Kolls JK Th17 cells and mucosal host defense. Semin Immunol (2007) 19:377–82. 10.1016/j.smim.2007.10.009 18054248 PMC2293278

[60] Dubin PJ Kolls JK. Th17 cytokines and mucosal immunity. Immunol Rev (2008) 226:160–71. 10.1111/j.1600-065X.2008.00703.x 19161423

[61] Raffatellu M Santos RL Verhoeven DE George MD Wilson RP Winter SE Simian immunodeficiency virus–induced mucosal interleukin-17 deficiency promotes *Salmonella* dissemination from the gut. Nat Med (2008) 14:421–8. 10.1038/nm1743 18376406 PMC2901863

[62] Lu Y-J Gross J Bogaert D Finn A Bagrade L Zhang Q Interleukin-17A mediates acquired immunity to pneumococcal colonization. PLoS Pathog (2008) 4:e1000159. 10.1371/journal.ppat.1000159 18802458 PMC2528945

