# Supplementary material for: T Helper 17 Promotes Induction of Antigen-Specific Gut-Mucosal Cytotoxic T Lymphocytes following Adenovirus Vector Vaccination
Source: Front Immunol. 2017 Nov 6;8:1456. doi: 10.3389/fimmu.2017.01456 (PMC5681732; doi:10.3389/fimmu.2017.01456)
Supplement: Supplementary file 1 [file Data_Sheet_1.pdf]

SUPPLEMENTARY MATERIAL 1

Supplementary Figure Legend

Supplementary Fig. 1. *Ifnar2*$^{-/-}$ CD4^{+} T cells differentiate normally into Th17 cells. Naïve CD4^{+} T cells from each mouse were cultured under Th17 differentiation conditions for 4 days, and the frequency of Th17 cells was determined according to the expression of IL-17A using flow cytometry. Data are representative of three independent experiments and are shown as the mean ± standard deviation (n = 3). *p < 0.05 (Student's t test).

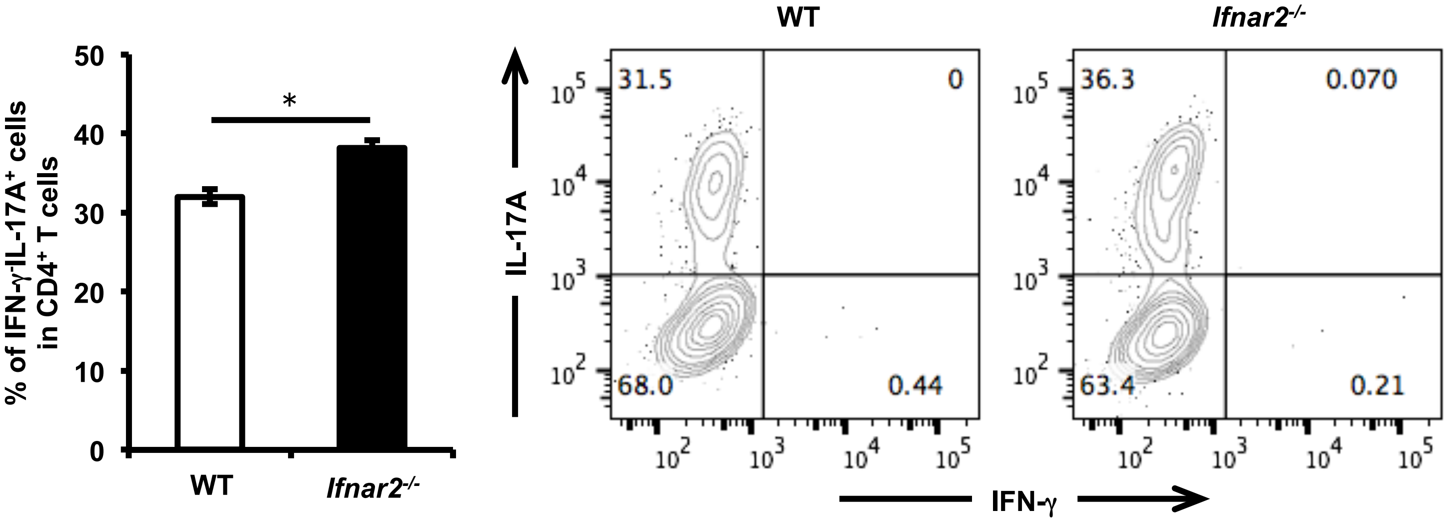

Supplementary Fig. 1 M. Hemmi *et al.*
