# Supplementary material for: T Helper 17 Promotes Induction of Antigen-Specific Gut-Mucosal Cytotoxic T Lymphocytes following Adenovirus Vector Vaccination
Source: Front Immunol. 2017 Nov 6;8:1456. doi: 10.3389/fimmu.2017.01456 (PMC5681732; doi:10.3389/fimmu.2017.01456)
Supplement: Supplementary file 2 [file Data_Sheet_2.pdf]

**SUPPLEMENTARY MATERIAL 2**

**Supplementary Figure Legend**

**Supplementary Fig. 2. cDCs, neutrophils, and macrophages are not regulated by type I IFN signaling. (A)** At 0, 8, 24, and 48 h after i.m. Adv vaccination, the frequency of Ly-6C$^{-}$MHC-II$^{+}$CD11c$^{hi}$ cDCs in iLNs was measured by flow cytometry. **(B)** At 24 h after vaccination, the frequency of Ly-6G$^{+}$CD11b$^{+}$ neutrophils and Ly-6G$^{-}$CD11b$^{+}$SSC$^{low}$ macrophages in CD45$^{+}$ cells from quadriceps muscles was measured by flow cytometry. Data are the pools of **(A)** four and **(B)** three independent experiments and are shown as the mean ± standard error of the mean [**(A)**: $n$ = 3–4; **(B)**: $n$ = 5–6]. *$p$ < 0.05; **$p$ < 0.01; ***$p$ < 0.001 [**(A)** Student's $t$ test; **(B)** one-way ANOVA].

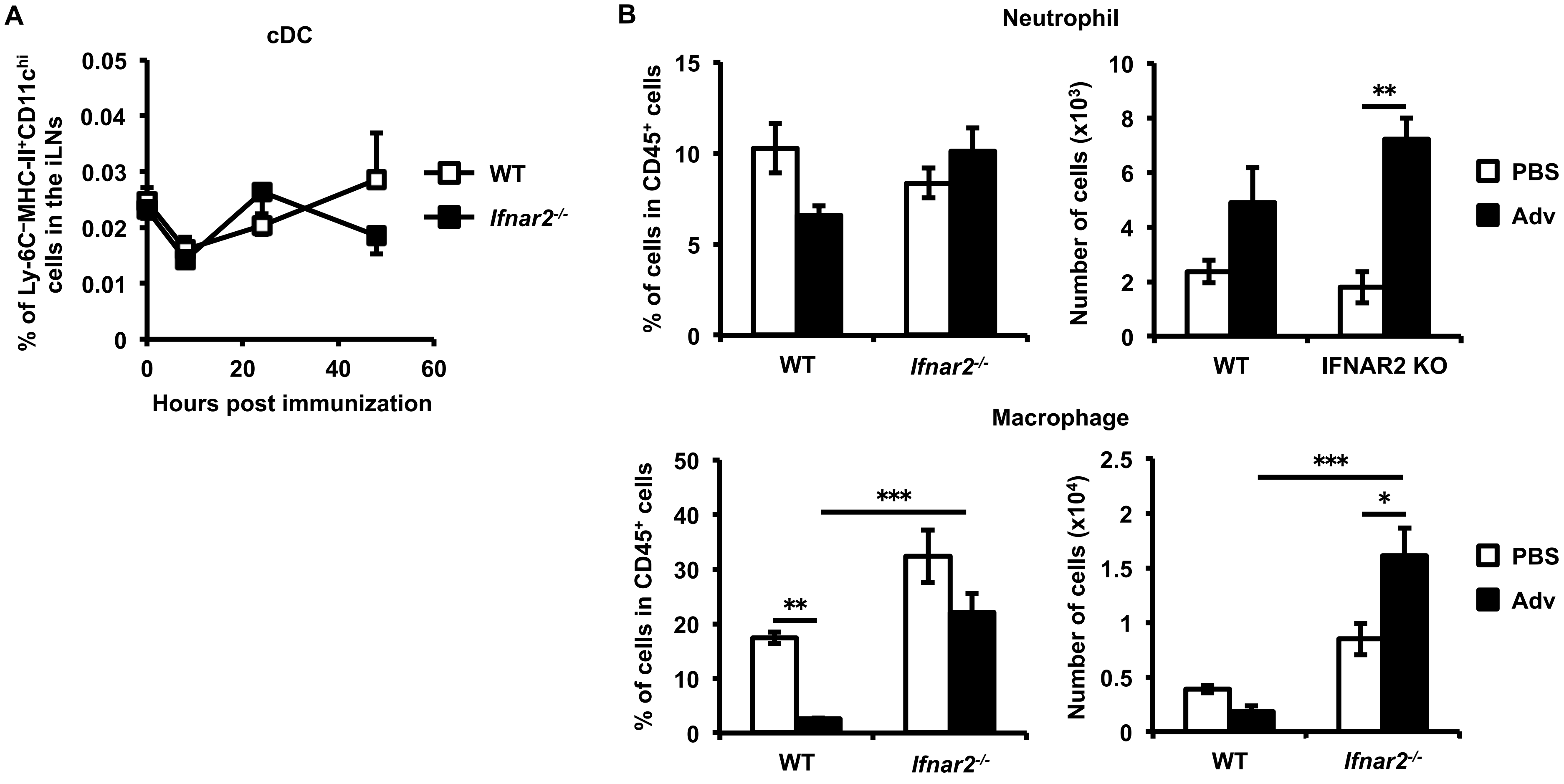


Supplementary Fig. 2 M. Hemmi *et al.*
